# Seven Hundred Album Verses

**Published:** 1886-10

**Authors:** 


### *CONSUMPTION CURABLE.*

In a treatise lately published by Prof. M. Jaccond, of Paris, with the above title, it is claimed that consumption may be cured at every stage. The author's conclusions are thus summarized: "The incurability proclaimed by Lænnec and his immediate successors is disproved by pathological anatomy and clinical observation. None should, therefore, allow themselves to be influenced by such an opinion, which is but an historical souvenir. When the existence of tubercles in the lungs is recognized it should not be inferred from that moment that he who has them is doomed to death because of their presence. Should it be found that the tubercles soften and a cavity forms, it should not be believed that all is lost. It has been shown that this is not the case, and the natural tendency which tubercle has to fibrous transmutation, that is, to recovery, should not be forgotten. Before being discouraged the physician should search and examine incessantly whether the patient is in the requisite conditions for such a favorable evolution. If all hope of absolute recovery must be abandoned, a relative cure should be wrought, and the attempt made to place the patient in such conditions that he can live, notwithstanding the lesions which are now irreparable; in a word, the plan adopted should be to strive, and to strive always, with the unshaken confidence that may be drawn from the notion that recovery is possible. The enemy can be conquered. This is the idea that should engender and sustain every effort. It is certain that this conviction is the first condition of success, since it is absence of faith in the possibility of a cure which prevents the adoption of good therapeutic treatment."

### *SEVEN HUNDRED ALBUM VERSES*

We have just received from the publishers a copy of a little book entitled, "Seven Hundred Album Verses," containing 128 pages of selections of prose and poetry, suitable for writing in autograph albums. If a majority of those who oblige their friends in this way would quote from this little book there would be an improvement in this species of literature, wherein all writers seem bound to court the muses, if not the fair one of the album. It is offered to be sent to any address by mail, in paper cover for 15 cents; cloth 30 cents, by J. S. Ogilvie & Co., publishers, 31 Rose Street, New York.

